# 2-[(*E*)-2-(4-Meth­oxy­phen­yl)ethen­yl]-1-methyl­pyridinium iodide

**DOI:** 10.1107/S1600536813031929

**Published:** 2013-11-30

**Authors:** K. Senthil, S. Kalainathan, A. RubanKumar, V. Ramkumar, Jiban Podder

**Affiliations:** a Centre for Crystal Growth, School of Advanced Sciences, VIT University, Vellore, Tamil Nadu, India; b Department of Chemistry, IIT Madras, Chennai 600 036, TamilNadu, India; c Department of Physics, Bangladesh University of Engineering and Technology, Dhaka 1000, Bangladesh

## Abstract

In the title mol­ecular salt, C_16_H_10_NO^+^·I^−^, the dihedral angle between the pyridinium and benzene rings is 6.61 (8)°. In the crystal, the cation is linked to the anion by a C—H⋯I inter­action arising from the activated aromatic C atom adjacent to the N^+^ cation.

## Related literature

For background to organic non-linear optical materials, see: Jagannathan *et al.* (2007 ▶); Williams (1984 ▶). For a related structure, see: Chantrapromma *et al.* (2010 ▶).
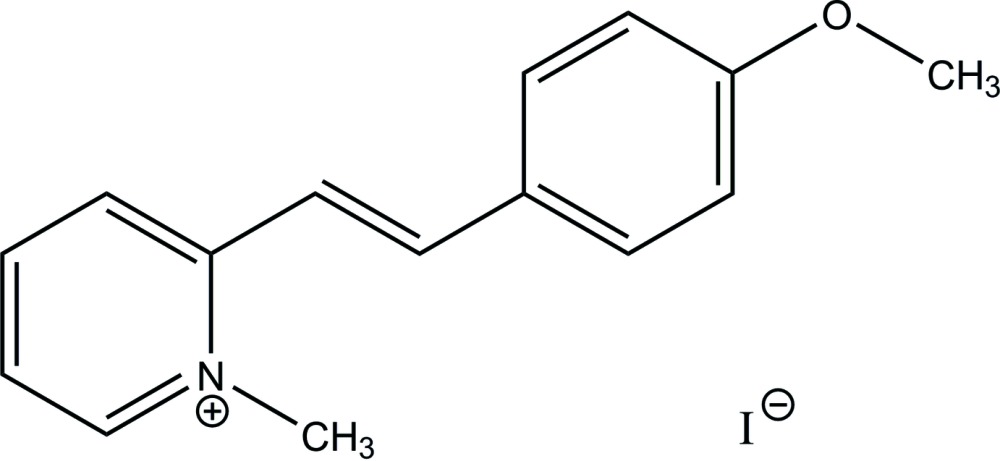


## Experimental

### 

#### Crystal data

C_15_H_16_NO^+^·I^−^
*M* *_r_* = 353.19
Triclinic, 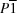

*a* = 7.1760 (3) Å
*b* = 8.6895 (4) Å
*c* = 12.1555 (6) Å
α = 92.645 (2)°
β = 92.115 (2)°
γ = 103.781 (2)°
*V* = 734.47 (6) Å^3^
*Z* = 2
Mo *K*α radiation
μ = 2.17 mm^−1^
*T* = 298 K
0.25 × 0.20 × 0.15 mm

#### Data collection

Bruker APEXII CCD diffractometer
Absorption correction: multi-scan (*SADABS*; Bruker, 2004 ▶) *T*_min_ = 0.613, *T*_max_ = 0.737
8523 measured reflections
2470 independent reflections
2363 reflections with *I* > 2σ(*I*)
*R*_int_ = 0.024

#### Refinement

*R*[*F* ^2^ > 2σ(*F* ^2^)] = 0.020
*wR*(*F* ^2^) = 0.052
*S* = 1.09
2470 reflections
165 parameters
H-atom parameters constrained
Δρ_max_ = 0.31 e Å^−3^
Δρ_min_ = −0.59 e Å^−3^

### 

Data collection: *APEX2* (Bruker, 2004 ▶); cell refinement: *SAINT* (Bruker, 2004 ▶); data reduction: *SAINT*; program(s) used to solve structure: *SHELXS97* (Sheldrick, 2008 ▶); program(s) used to refine structure: *SHELXL97* (Sheldrick, 2008 ▶); molecular graphics: *ORTEP-3 for Windows* (Farrugia, 2012 ▶); software used to prepare material for publication: *SHELXL97*.

## Supplementary Material

Crystal structure: contains datablock(s) global, I. DOI: 10.1107/S1600536813031929/hb7162sup1.cif

Structure factors: contains datablock(s) I. DOI: 10.1107/S1600536813031929/hb7162Isup2.hkl

Supplementary material file. DOI: 10.1107/S1600536813031929/hb7162Isup3.cml

Additional supplementary materials:  crystallographic information; 3D view; checkCIF report

## Figures and Tables

**Table 1:** Hydrogen-bond geometry (Å, °)

*D*—H⋯*A*	*D*—H	H⋯*A*	*D*⋯*A*	*D*—H⋯*A*
C1—H1⋯I1^i^	0.93	2.96	3.872 (3)	168

Symmetry code: (i) 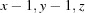.

**Fig. 1.**
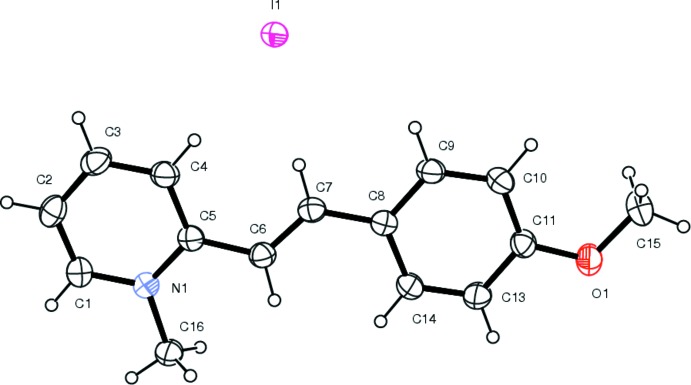
ORTEP of the molecule with atoms represented as 30% probability ellipsoids.

## supplementary crystallographic information

### 1. Comment

In recent years, the design of new organic nonlinear optical (NLO) materials have been studied (e.g. Jagannathan *et al.*, 2007). As part of our stuies in this area, the title pyridinium derivative compound was synthesized,. It crystallizes in the centrosymmetric Pī triclinic space group, so it does not exhibit second-order nonlinear optical properties (Williams, 1984).

The cation is essentially planar and exist in E configuration. The dihedral angle between the pyridinium and benzene rings is 6.16 (8)°. Bond lengths and angles are comparable with those for closely related structure (Chantrapromma *et al.*, 2010). In the crystal, the cation is linked to the anion by a C—H···I interaction (Table 1).

### 2. Experimental

The title compound was prepared by mixing 1:1 molar ratio of solutions of 1,2-dimethylpyridinium iodide (7.052 g, 30 mmol), 4-methoxy benzaldehyde (3.7 ml, 30 mmol) and piperidine (5 drops) in hot methanol (20 ml). The resulting mixture was refluxed at 60°C for 8 h to give yellowish crystalline product, which was filtered off and washed with diethyl ether and dried. Yellow needle-shaped single crystals of the title compound suitable for X-ray structure determination were obtained by recrystallization (three times) from methanol–acetonitrile (1:1) mixture by slow evaporation of the solvent at ambient temperature over several days (m.p. 514–516 K).

### 3. Refinement

All hydrogen atoms were fixed geometrically (C—H 0.93–0.98 Å) and refined as riding, with *U*_iso_(H) = 1.2–1.5 *U*_eq_(C).

### Crystal data

**Table:**

C_15_H_16_NO^+^·I^−^	*Z* = 2
*M**_r_* = 353.19	*F*(000) = 348
Triclinic, *P*1	*D*_x_ = 1.597 Mg m^−^^3^
Hall symbol: -P 1	Mo *K*α radiation, λ = 0.71073 Å
*a* = 7.1760 (3) Å	Cell parameters from 8234 reflections
*b* = 8.6895 (4) Å	θ = 2.4–28.4°
*c* = 12.1555 (6) Å	µ = 2.17 mm^−^^1^
α = 92.645 (2)°	*T* = 298 K
β = 92.115 (2)°	Slab, yellow
γ = 103.781 (2)°	0.25 × 0.20 × 0.15 mm
*V* = 734.47 (6) Å^3^	

### Data collection

**Table:**

Bruker APEXII CCD diffractometer	2470 independent reflections
Radiation source: fine-focus sealed tube	2363 reflections with *I* > 2σ(*I*)
Graphite monochromator	*R*_int_ = 0.024
phi and ω scans	θ_max_ = 25.0°, θ_min_ = 1.7°
Absorption correction: multi-scan (*SADABS*; Bruker, 2004)	*h* = −8→6
*T*_min_ = 0.613, *T*_max_ = 0.737	*k* = −10→10
8523 measured reflections	*l* = −12→14

### Refinement

**Table:**

Refinement on *F*^2^	Secondary atom site location: difference Fourier map
Least-squares matrix: full	Hydrogen site location: inferred from neighbouring sites
*R*[*F*^2^ > 2σ(*F*^2^)] = 0.020	H-atom parameters constrained
*wR*(*F*^2^) = 0.052	*w* = 1/[σ^2^(*F*_o_^2^) + (0.0211*P*)^2^ + 0.414*P*] where *P* = (*F*_o_^2^ + 2*F*_c_^2^)/3
*S* = 1.09	(Δ/σ)_max_ = 0.001
2470 reflections	Δρ_max_ = 0.31 e Å^−^^3^
165 parameters	Δρ_min_ = −0.59 e Å^−^^3^
0 restraints	Extinction correction: *SHELXL97* (Sheldrick, 2008), Fc^*^=kFc[1+0.001xFc^2^λ^3^/sin(2θ)]^-1/4^
Primary atom site location: structure-invariant direct methods	Extinction coefficient: 0.0288 (14)

### Special details

**Table:**

Geometry. All e.s.d.'s (except the e.s.d. in the dihedral angle between two l.s. planes) are estimated using the full covariance matrix. The cell e.s.d.'s are taken into account individually in the estimation of e.s.d.'s in distances, angles and torsion angles; correlations between e.s.d.'s in cell parameters are only used when they are defined by crystal symmetry. An approximate (isotropic) treatment of cell e.s.d.'s is used for estimating e.s.d.'s involving l.s. planes.
Refinement. Refinement of *F*^2^ against ALL reflections. The weighted *R*-factor *wR* and goodness of fit *S* are based on *F*^2^, conventional *R*-factors *R* are based on *F*, with *F* set to zero for negative *F*^2^. The threshold expression of *F*^2^ > σ(*F*^2^) is used only for calculating *R*-factors(gt) *etc*. and is not relevant to the choice of reflections for refinement. *R*-factors based on *F*^2^ are statistically about twice as large as those based on *F*, and *R*- factors based on ALL data will be even larger.

### Fractional atomic coordinates and isotropic or equivalent isotropic displacement parameters (Å^2^)

**Table:**

	*x*	*y*	*z*	*U*_iso_*/*U*_eq_	
C1	−0.1445 (4)	0.0480 (3)	0.8878 (2)	0.0494 (6)	
H1	−0.2582	−0.0296	0.8742	0.059*	
C2	−0.1376 (4)	0.1673 (3)	0.9651 (2)	0.0560 (7)	
H2	−0.2451	0.1722	1.0043	0.067*	
C3	0.0321 (5)	0.2809 (3)	0.9843 (2)	0.0598 (7)	
H3	0.0397	0.3633	1.0372	0.072*	
C4	0.1896 (4)	0.2736 (3)	0.9260 (2)	0.0537 (6)	
H4	0.3039	0.3503	0.9400	0.064*	
C5	0.1799 (4)	0.1513 (3)	0.84555 (19)	0.0419 (5)	
C6	0.3386 (4)	0.1382 (3)	0.7790 (2)	0.0468 (6)	
H6	0.3297	0.0432	0.7383	0.056*	
C7	0.4961 (4)	0.2531 (3)	0.7722 (2)	0.0460 (5)	
H7	0.5055	0.3449	0.8167	0.055*	
C8	0.6550 (3)	0.2503 (3)	0.7027 (2)	0.0422 (5)	
C9	0.8189 (4)	0.3743 (3)	0.7121 (2)	0.0497 (6)	
H9	0.8233	0.4583	0.7632	0.060*	
C10	0.9754 (4)	0.3779 (3)	0.6488 (2)	0.0497 (6)	
H10	1.0838	0.4618	0.6580	0.060*	
C11	0.9687 (4)	0.2554 (3)	0.5718 (2)	0.0497 (6)	
C13	0.8062 (4)	0.1296 (3)	0.5607 (3)	0.0622 (7)	
H13	0.8019	0.0465	0.5088	0.075*	
C14	0.6536 (4)	0.1264 (3)	0.6246 (2)	0.0549 (6)	
H14	0.5470	0.0408	0.6163	0.066*	
C15	1.2825 (4)	0.3695 (4)	0.5113 (3)	0.0652 (8)	
H15A	1.2501	0.4670	0.4943	0.098*	
H15B	1.3708	0.3459	0.4594	0.098*	
H15C	1.3410	0.3800	0.5845	0.098*	
C16	−0.0104 (4)	−0.0956 (3)	0.7487 (2)	0.0561 (7)	
H16A	−0.1383	−0.1617	0.7491	0.084*	
H16B	0.0805	−0.1563	0.7676	0.084*	
H16C	0.0128	−0.0567	0.6766	0.084*	
N1	0.0107 (3)	0.0400 (2)	0.83019 (16)	0.0409 (4)	
O1	1.1126 (3)	0.2441 (3)	0.50511 (19)	0.0726 (6)	
I1	0.43035 (2)	0.689767 (18)	0.803640 (16)	0.05847 (11)	

### Atomic displacement parameters (Å^2^)

**Table:**

	*U*^11^	*U*^22^	*U*^33^	*U*^12^	*U*^13^	*U*^23^
C1	0.0449 (13)	0.0580 (14)	0.0471 (14)	0.0149 (11)	0.0023 (11)	0.0090 (11)
C2	0.0638 (17)	0.0679 (16)	0.0428 (14)	0.0267 (14)	0.0105 (12)	0.0086 (12)
C3	0.085 (2)	0.0533 (15)	0.0434 (14)	0.0208 (14)	0.0134 (14)	0.0002 (11)
C4	0.0653 (16)	0.0454 (13)	0.0461 (14)	0.0048 (12)	0.0071 (12)	0.0004 (11)
C5	0.0502 (13)	0.0407 (11)	0.0355 (12)	0.0111 (10)	0.0015 (10)	0.0082 (9)
C6	0.0512 (14)	0.0437 (12)	0.0445 (13)	0.0096 (11)	0.0032 (11)	−0.0003 (10)
C7	0.0524 (14)	0.0402 (12)	0.0460 (14)	0.0123 (10)	0.0012 (11)	0.0025 (10)
C8	0.0435 (12)	0.0410 (11)	0.0425 (13)	0.0114 (10)	−0.0032 (10)	0.0045 (9)
C9	0.0563 (15)	0.0415 (12)	0.0475 (14)	0.0053 (11)	0.0016 (12)	−0.0025 (10)
C10	0.0460 (13)	0.0471 (13)	0.0510 (15)	0.0022 (10)	−0.0012 (11)	0.0014 (11)
C11	0.0401 (13)	0.0570 (14)	0.0507 (15)	0.0100 (11)	−0.0010 (11)	−0.0012 (11)
C13	0.0506 (15)	0.0616 (16)	0.0677 (19)	0.0053 (13)	0.0024 (13)	−0.0226 (14)
C14	0.0422 (13)	0.0537 (14)	0.0620 (17)	0.0012 (11)	−0.0002 (12)	−0.0104 (12)
C15	0.0443 (15)	0.0802 (19)	0.0671 (19)	0.0053 (14)	0.0059 (13)	0.0111 (15)
C16	0.0487 (14)	0.0548 (14)	0.0596 (17)	0.0057 (12)	0.0012 (12)	−0.0122 (12)
N1	0.0442 (11)	0.0444 (10)	0.0349 (10)	0.0125 (8)	−0.0016 (8)	0.0045 (8)
O1	0.0491 (11)	0.0816 (14)	0.0795 (15)	0.0041 (10)	0.0153 (10)	−0.0219 (11)
I1	0.04875 (13)	0.04564 (12)	0.07815 (17)	0.00721 (8)	0.00877 (9)	−0.00920 (8)

### Geometric parameters (Å, º)

**Table:**

C1—N1	1.351 (3)	C9—C10	1.380 (4)
C1—C2	1.357 (4)	C9—H9	0.9300
C1—H1	0.9300	C10—C11	1.375 (4)
C2—C3	1.377 (4)	C10—H10	0.9300
C2—H2	0.9300	C11—O1	1.355 (3)
C3—C4	1.369 (4)	C11—C13	1.393 (4)
C3—H3	0.9300	C13—C14	1.362 (4)
C4—C5	1.397 (4)	C13—H13	0.9300
C4—H4	0.9300	C14—H14	0.9300
C5—N1	1.361 (3)	C15—O1	1.426 (3)
C5—C6	1.445 (3)	C15—H15A	0.9600
C6—C7	1.326 (4)	C15—H15B	0.9600
C6—H6	0.9300	C15—H15C	0.9600
C7—C8	1.448 (4)	C16—N1	1.479 (3)
C7—H7	0.9300	C16—H16A	0.9600
C8—C9	1.390 (3)	C16—H16B	0.9600
C8—C14	1.400 (4)	C16—H16C	0.9600
			
N1—C1—C2	121.2 (2)	C11—C10—H10	120.5
N1—C1—H1	119.4	C9—C10—H10	120.5
C2—C1—H1	119.4	O1—C11—C10	124.9 (2)
C1—C2—C3	118.6 (3)	O1—C11—C13	115.6 (2)
C1—C2—H2	120.7	C10—C11—C13	119.5 (2)
C3—C2—H2	120.7	C14—C13—C11	121.0 (3)
C4—C3—C2	120.5 (3)	C14—C13—H13	119.5
C4—C3—H3	119.8	C11—C13—H13	119.5
C2—C3—H3	119.8	C13—C14—C8	120.9 (2)
C3—C4—C5	120.4 (3)	C13—C14—H14	119.6
C3—C4—H4	119.8	C8—C14—H14	119.6
C5—C4—H4	119.8	O1—C15—H15A	109.5
N1—C5—C4	117.4 (2)	O1—C15—H15B	109.5
N1—C5—C6	119.1 (2)	H15A—C15—H15B	109.5
C4—C5—C6	123.5 (2)	O1—C15—H15C	109.5
C7—C6—C5	124.1 (2)	H15A—C15—H15C	109.5
C7—C6—H6	117.9	H15B—C15—H15C	109.5
C5—C6—H6	117.9	N1—C16—H16A	109.5
C6—C7—C8	126.7 (2)	N1—C16—H16B	109.5
C6—C7—H7	116.6	H16A—C16—H16B	109.5
C8—C7—H7	116.6	N1—C16—H16C	109.5
C9—C8—C14	117.0 (2)	H16A—C16—H16C	109.5
C9—C8—C7	120.0 (2)	H16B—C16—H16C	109.5
C14—C8—C7	123.0 (2)	C1—N1—C5	121.9 (2)
C10—C9—C8	122.7 (2)	C1—N1—C16	117.2 (2)
C10—C9—H9	118.7	C5—N1—C16	120.9 (2)
C8—C9—H9	118.7	C11—O1—C15	118.6 (2)
C11—C10—C9	119.0 (2)		
			
N1—C1—C2—C3	−0.2 (4)	C9—C10—C11—C13	−1.1 (4)
C1—C2—C3—C4	0.3 (4)	O1—C11—C13—C14	178.9 (3)
C2—C3—C4—C5	0.6 (4)	C10—C11—C13—C14	0.2 (5)
C3—C4—C5—N1	−1.5 (4)	C11—C13—C14—C8	0.6 (5)
C3—C4—C5—C6	178.4 (2)	C9—C8—C14—C13	−0.6 (4)
N1—C5—C6—C7	167.1 (2)	C7—C8—C14—C13	179.6 (3)
C4—C5—C6—C7	−12.8 (4)	C2—C1—N1—C5	−0.9 (4)
C5—C6—C7—C8	−176.5 (2)	C2—C1—N1—C16	179.3 (2)
C6—C7—C8—C9	−174.0 (3)	C4—C5—N1—C1	1.7 (3)
C6—C7—C8—C14	5.8 (4)	C6—C5—N1—C1	−178.2 (2)
C14—C8—C9—C10	−0.2 (4)	C4—C5—N1—C16	−178.4 (2)
C7—C8—C9—C10	179.5 (2)	C6—C5—N1—C16	1.6 (3)
C8—C9—C10—C11	1.1 (4)	C10—C11—O1—C15	−2.1 (4)
C9—C10—C11—O1	−179.6 (3)	C13—C11—O1—C15	179.4 (3)

### Hydrogen-bond geometry (Å, º)

**Table:**

*D*—H···*A*	*D*—H	H···*A*	*D*···*A*	*D*—H···*A*
C1—H1···I1^i^	0.93	2.96	3.872 (3)	168

Symmetry code: (i) *x*−1, *y*−1, *z*.

## References

[bb1] Bruker (2004). *APEX2*, *SAINT* and *SADABS* Bruker AXS Inc., Madison, Wisconsin, USA.

[bb2] Chantrapromma, S., Chanawanno, K. & Fun, H.-K. (2010). *Acta Cryst.* E**66**, o1975–o1976. 10.1107/S1600536810026309 PMC3007387 21588293

[bb3] Farrugia, L. J. (2012). *J. Appl. Cryst.* **45**, 849–854.

[bb4] Jagannathan, K., Kalainathan, S., Gnanasekaran, T., Vijayan, N. & Bhagavan­narayana, G. (2007). *Cryst. Res. Technol.* **42**, 483–487.

[bb5] Sheldrick, G. M. (2008). *Acta Cryst.* A**64**, 112–122. 10.1107/S0108767307043930 18156677

[bb6] Williams, D. J. (1984). *Angew. Chem. Int. Ed. Engl.* **23**, 690–703.

